# The Medical, Surgical and Hygienic Exhibition

**Published:** 1903-06-13

**Authors:** 


### THE MEDICAL, SURGICAL AND HYGIENIC EXHIBITION.

The object of the Medical, Surgical and Hygienic Exhibitors' Association, Limited, is to provide an effective and convenient means by which medical men and others can ascertain the progress that has been made in the manufacture of preparations and appliances relating to medicine, surgery, dietetics, hygiene, and allied branches, and the annual exhibition, which was held on June 2nd, 3rd, 4th, and 5th, at the Queen's Hall, Langham Place, provided ample evidence of the continued success of the scheme by the increase both in the number of exhibitors and in the attendance of medical men, trained nurses, and officials of hospitals and infirmaries. This year food products were unusually numerous. Karnoid, Limited, showed their whole-meat preparations of that name, the nutritive value of which renders them well adapted for invalid use. The highly concentrated fluid beef "Oxo" was the principal item on the stall of Liebig's Extract of Meat Company. The Shredded Wheat Company exhibited among their well-known whole-wheat preparations the new product, "Triscuit," which is pure whole wheat in wafer form, and Oederlein's Swiss milk vermicelli, a nourishing carbohydrate food for children. The malted cereal foods of Telma, Limited, were on view, as also were the nutritious milk proteid manufactures of the Protene Company, Limited. Vegox, which is a convenient preparation of beef essence and vegetables, was prominent, while "Bovril" in various forms and "Virol," the well-known red bone marrow food, are entitled to special mention. Boyd's Banana Malted Food, which attracted much notice, was shown by the Medical and General Specialities Company, and Boyd's antiseptic perfumes and toilet requisites were on the same stall. Maggi's Consommé, Tonic Bouillon, soups, etc., commendable for their nourishing properties and agreeable flavour, were shown by Cozensa and Co. Maltova, a concentrated preparation of eggs and malt of high excellence, was shown by Maltova, Limited, and the Condensed Egg Syndicate exhibited a similar preparation of undoubted value called Ovumalt. Messrs. G. Van Abbott and Sons exhibited bread and biscuit foods in much variety for diabetics, and food preparations for dyspeptics, invalids, and delicate children. Messrs. Callard and Co. introduced several palatable new foods for diabetes and obesity, and the Manhu Food Company exhibited the Manhu diabetic specialities. With Cadbury's Cocoa, the name of which suffices to commend it, was shown a milk chocolate of exquisite taste. Nestlé's Food for Infants and Invalids, and the condensed milk products of Mr. Henri Nestlé, which are deservedly held in universal favour, received marked attention. Cerebos Salt for table and culinary use (a great improvement on common salt) was shown, while the Salt Union, Limited, exhibited an excellent new table salt, also Droitwich Brine Crystals, for employment in the administration of brine baths. The principal exhibit of the Peptenzyme Company was a preparation named Peptenzyme, which has been found by investigation to be an effective digestant. Messrs. Cooper and Co. had an attractive display of oxycarbonated mineral waters in porcelain-lined syphons, while Rosbach, a natural mineral water of unquestionable excellence, was well in evidence.

Turning to drugs, Mr. W. Martindale contributed an extensive exhibit of medical appliances and preparations, and a new antiseptic and disinfectant called Lysoform, which is eminently suitable for disinfecting instruments, wounds, etc. The Denver Chemical Manufacturing Co. exhibited Antiphlogistine, which is an antiseptic and absorbent dressing, in paste form, efficacious in local inflammatory affections. Ovo Lecithin (Billon's Ovo Lecithin Dépôt) received particular attention for its properties as a phosphoric medicine. Messrs. Benque and Co., of Paris, exhibited an anæsthetic called Narcotile (a pure ether and chemical combination) and an apparatus for its administration. Leslie's, Limited, with their tape plasters, antiseptic dressings (prepared according to the formulæ of Lord Lister), absorbent lint and Geneva crêpe bandage, gave a most important exhibit, and the Sanitary Wood Wool Co. Limited, also attracted much attention with Hartmann's antiseptic wood wool preparations. The Izal disinfectants, which are now universally employed, and the medical and toilet preparations bearing that name, were conspicuous, as also was Wright's coal tar soap, so well known in the nursery, and Liquor Carbonis Detergens, the superiority of which is established. The Oowana toilet soap, which is prepared from white Castille, deliciously scented, and can be used on the most delicate skin, claims special notice. The comprehensive display of instruments and requisites for surgical and nursing purposes, as shown by Messrs. Garrould, had marked interest for nurses.

With regard to literature, the Scientific Press, Limited, had on view a number of works on medical subjects, hygiene, standard books on hospitals, and text books on nursing in its various branches.

The Berkefeld Filter Company, Limited, invited medical criticism on their filters, which are simple in construction, easily cleaned, and in which the work is done by cylinders made of Kieselguhr, which is a finely porous substance. The Rapid Tap Filter (Lehmann's system) was much admired for its simplicity and durability, while the Pasteur (Chamberland) Filter, as shown by Messrs. J. Defries and Sons, Limited, which has been extensively employed by the Government, drew considerable attention.

The Empire bath cabinet deserves commendation for the rapidity with which ingress and egress can be effected, and for its cheapness, while the Gem bath cabinet, which is well known, received considerable attention. An admirable hospital ward stove, by Messrs. Hendry and Pattisson, so constructed as to secure ventilation by warm fresh air, was much approved and should secure many patrons.

The baths and appliances of the Dowsing Radiant Heat Company, which have been so successfully employed in the treatment of rheumatism, gout and kindred diseases, were in operation and were a general attraction. Another important feature of the exhibition was the exhibit of Messrs. Doulton and Co., which included sanitary fittings specially designed for institutions and operating lavatories, the valves of which can be worked by the elbow, leaving the hands free in accordance with modern aseptic principles.

